# Is non-union of tibial shaft fractures due to nonculturable bacterial pathogens? A clinical investigation using PCR and culture techniques

**DOI:** 10.1186/1749-799X-7-20

**Published:** 2012-05-20

**Authors:** Justus Gille, Steffen Wallstabe, Arndt-Peter Schulz, Andreas Paech, Ulf Gerlach

**Affiliations:** 1 Department of Trauma and Reconstructive Surgery, University of Schleswig-Holstein, Campus Luebeck, Luebeck, 23538, Germany; 2 Department of Trauma Surgery and Sportsmedicine, BG-Traumahospital Hamburg, Hamburg, 21033, Germany

**Keywords:** Non-union, Tibial fracture, Low-grade infection, Molecular diagnosis, PCR technique

## Abstract

**Background:**

Non-union continues to be one of the orthopedist’s greatest challenges. Despite effective culture methods, the detection of low-grade infection in patients with non-union following tibial fracture still presents a challenge. We investigated whether “aseptic” tibial non-union can be the result of an unrecognized infection.

**Methods:**

A total of 23 patients with non-union following tibial shaft fractures without clinical signs of infection were investigated. Intraoperative biopsy samples obtained from the non-union site were examined by means of routine culture methods and by polymerase chain reaction (PCR) for the detection of 16 S ribosomal RNA (rRNA). Control subjects included 12 patients with tibial shaft fractures.

**Results:**

23 patients (8 women and 15 men; mean age: 47.4 years) were included into this study. Preoperative C-reactive protein levels (mean: 20.8 mg/l) and WBC counts (mean: 8,359/μl) in the study group were not significantly higher than in the control group. None of the samples of non-union routine cultures yielded microorganism growth. Bacterial isolates were found by conventional culturing methods in only 1 case of an open fracture from the control group. In this case, PCR yielded negative results. 16 S rRNA was detected in tissue specimens from 2 patients (8.7%) with non-union. The analysis of these variable species-specific sequences enabled the identification of specific microorganisms (1x *Methylobacterium* species, 1x *Staphylococcus* species). Both PCR-positive patients were culture-negative.

**Conclusions:**

The combination of microbiological culture and broad-range PCR seems to substantially add to the number of microbiological diagnoses obtained and may improve the clinican’s ability to tailor therapy to the individual patient’s needs.

## Article summary

We investigated whether “aseptic” tibial non-union is related to unrecognized infection. 23 patients (8 women and 15 men; mean age: 47.4 years) were included in the study. 16 S rRNA was detected in 2 cases (8.7%) of non-union. PCR-positive patients were culture-negative in both cases.

## Introduction

Diaphyseal tibia fractures are the most common lower limb fractures worldwide [1]. Despite advances in management, tibia fractures remain vulnerable to many complications, which often require secondary surgery. Potential complications include delayed union, non-union, malunion, compartment syndrome and infection [2]. A recent study on open and closed diaphyseal tibia fractures treated by all modalities reported an overall revision rate of 22.4% [3], which was often the result of non-unions.

In about 10% of cases the healing process is delayed [4]; for certain at-risk patients, it can affect over 30% [5]. Established causes of delayed union and non-union of tibial fractures are systemic deficits, e.g. advanced patient age [6] and diabetes mellitus [7]; prior local impairment of the extremity, e.g. chronic impairment of the soft tissues or blood circulation [8]; and characteristics involving the traumatic impact itself, e.g. fracture localization [9], degree of soft tissue damage [10] and bacterial contamination [1,11].

Non-union, especially when infected, continues to be one of the greatest challenges in orthopedic surgery. After open reduction and internal fixation of tibial shaft fractures, the rate of superficial infection is up to 22% and deep infections occur in up to 15% [2], the latter of which can potentially lead to septic nonunion of the tibia. Clinical signs, laboratory investigations of infection parameters and microbiological findings are often insufficient for detecting infection. Distinguishing between infection and aseptic non-union is essential for determining the proper clinical course of action [12]. The standard analyses for detecting microorganisms – gram staining (for microscopic investigation) and culturing of tissue biopsy specimens obtained during surgical revision – are reported to have poor sensitivity [13]. The hypothesis is that evidence of bacterial infection is supported further by the detection of bacterial DNA, which suggests bacterial persistence in the area of non-union despite negative culture results. PCR targeting highly conserved regions of the bacterial genome (e.g. the 16 S rRNA gene) has been used successfully to detect nonculturable bacteria that cause a variety of infections, including septic arthritis [14] and meningitis [15]. The aim of this study was to investigate whether”aseptic” nonunion after tibial shaft fractures is due to nonculturable bacteria by means of PCR amplification of 16 S rRNA genes and to compare the efficiency of PCR with that of standard culture techniques.

## Materials and methods

All patients participating in the present study were informed in detail about the surgical technique and all alternative procedures with their respective advantages and disadvantages, and all participants chose to undergo the index surgical procedure. All patients signed informed consent forms to participate in the study. The study was performed in accordance with the local ethical review board.

**Figure 1:**
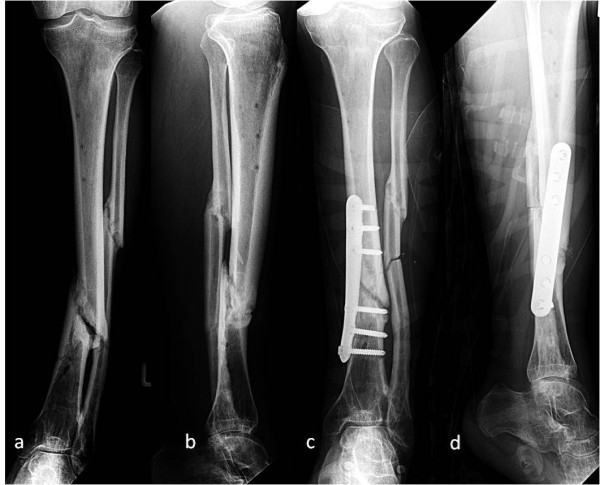
**Consecutive x-rays of a 30-year-old male with tibial non-union and valgus malalignment following stabilization with external fixator for 6 months (Figures a,b)**. Postoperative findings after excision of necrotic tissue and re-osteosynthesis with a multidirectional locking plate (tifix®-tibia-plate, Litos, Hamburg, Germany) in combination with reconstruction of skeletal defects by implantation of autologous bone removed from the iliac crest (Figures c,d). Conventional and molecular bacteria detection methods were both negative.

From November 2009 through March 2010, a consecutive series of 23 patients with non-union following tibial shaft fractures were investigated. Normal healing was defined as union within 4 months, delayed union as healing between 4 and 6 months and non-union was defined as the absence of healing after 6 months [16]. Only patients without clinical signs of infection were included in the study. Exemplary x-rays of one case from the treatment group are shown in Figure 1. Control subjects included 12 patients undergoing open reduction and internal fixation for acute tibial shaft fractures.

A stage-adapted treatment algorithm for tibial non-union has been established, as previously published [16]. Based on contemporary evidence among the recent literature, we do not routinely administer antibiotics beyond 5 days postoperatively [17]. We favor oral antibiotic administration, because our experience has shown us that the route of antibiotic administration (oral versus parenteral) does not affect the rate of disease remission if the bacteria are sensitive to the antibiotic used.

All patients received standard preoperative care. Skin was decontaminated with Cutasept H (Bode Chemie, Germany). Two grams of cefazolin (Basocef, Deltaselect GmbH, Germany) were administered for perioperative prophylaxis subsequent to taking the biopsies. Intraoperative biopsy samples (at least 3, each measuring 1 cm^3^) obtained from the non-union were all divided into 2 portions. Specimens were placed into separate sterile tubes without additional substrates. Specimens obtained for PCR were stored at −70°C. Samples were then examined by means of routine culture methods and by polymerase chain reaction (PCR) for the detection of 16 S ribosomal RNA (rRNA) in the laboratory. These procedures permitted the independent examination and interpretation of the results. Histopathologic findings were not recorded because of poor sensitivity, especially in cases of low-grade infection [18,19].

### Bacterial isolation and standard culture methods

All specimens were incubated in brain-heart infusion broth (bioMérieux, Nuertingen, Germany), TVLS medium [20], a medium described by Lodenkaemper and Stinen [21], on Columbia blood plates (aerobic 5% CO_2_), and then on Brucella agar plates (anaerobic) (bioMérieux, Nuertingen, Germany), as previously described [12]. Samples were incubated for 14 days. Susceptibility testing was performed according to the German Institutional Standard Nr. 58940 [22].

### DNA isolation

Tissue samples were immediately stored at −70°C, and DNA was purified from homogenized specimens after proteinase K digestion and column extraction with the NucleoSpin DNA kit (Macherey-Nagel, Dueren, Germany). All DNA procedures before and after PCR were performed in separate designated rooms with separate pipetting devices to avoid contamination of the samples with foreign DNA. Master-mixture water controls were used for every sample that was processed.

### PCR amplification

The sequences of the universal primers (16rRNA gene) and primers of the control gene (glyceraldehydes-3-phosphate dehydrogenase; GAPDH) are indicated in Table 1. Oligonucleotides used in this study were provided by Eurofins MWG Operon, Ebersberg, Germany. DNA was amplified in a 25μL-reaction mixture consisting of ready-to-go PCR beads (up to 23μL; Amersham Pharmacia Biotech, Muenchen, Germany), 0.5μL of each primer (100 pmol/mL), and 1μL of the sample. After amplification, 5μL of the amplified product was analyzed in a 2% agarose gel. Amplification products were sequenced by SeqLab and then analyzed using the National Center for Biotechnology Investigation Blast database [23,24].

**Table 1:** Nucleotide sequences of primers used to determine agents responsible for non-union following tibial shaft fractures

**Primer**	**Sequence**
**16S rRNA**	
**Forward**	5′-AGAGTTTGATCCTGGCTCAG-3′
**Reverse**	5′-CCCACTGCTGCCCGTAG-3′
**GAPDH**	
**Forward**	5′-TCTGCCCCCTCTGCTGATGCCCCC-3′
**Reverse**	5′-CCATCACGCCACAGTTTCCCGGAG-3′
**Note. GAPDH**	Glyceraldehydes-3-phosphate dehydrogenase

Amplification of the GAPDH control gene was performed using real-time PCR (Light Cycler Detection System; Roche Molecular Biochemicals, Mannheim, Germany) with the FastStart DNA Master SYBR kit (Roche Molecular Biochemicals). The PCR protocol included the following work stages: 95°C for 10 min, followed by 40 cycles at 95°C for 10 s, 60°C for 5 s, and 72°C for 10s. In the dissociation protocol, single peaks were confirmed to exclude nonspecific amplification.

### Statistical analysis

Statistical analysis was performed with the Statistical Package for the Social Sciences (SPSS 15.0, Chicago, IL, USA) for descriptive statistics with a level of significance set at *p* < 0.05. The non-parametric Wilcoxon’s signed rank test was used to analyze the data.

## Results

The experimental group of patients with tibial non-union consisted of 8 women and 15 men, with a mean age of 47.4 years (range: 20–82 years). The mean age of the control group was 49.3 years (range: 17–73 years), including 2 females and 10 males.

The mean interval of the fracture to the index operation due to non-union was 10.2 months (range: 6–34 months). At the time of the initial trauma, 8 cases (34.8%) were rated as open fractures due to severe soft tissue injuries according to the classification by Gustilo et al. [25]. Four open fractures (33.3%) were counted in the control group. Up to 8 revision surgeries (mean: 1.9; range: 1–8) had been performed in 18 cases (78.3%) prior to the index operation.

For all patients in the experimental group, clinical pathological data – especially C-reactive protein levels (mean: 20.8 mg/l; range: 1.6–92.7) and WBC counts (8,359/μl; range: 4,360–15,130) were not significantly higher compared to the control group (CRP: mean: 25.7 mg/l; range: 2.9–133, WBC: mean: 11,234/μl; range: 6,110–17,210).

**Figure 2:**
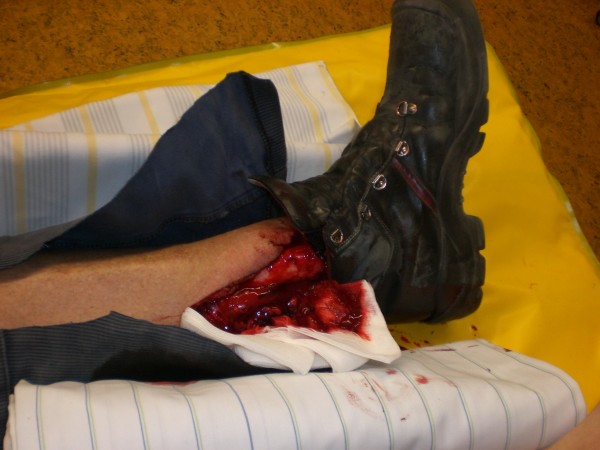
**38-year-old male with an open tibial fracture (control group)**. This figure demonstrates the findings before the initial treatment. The isolated pathogens in culture were *Streptococcus suis* and *Enterococcus* species. Tissue from the fracture gap after initial débridement yielded negative results by PCR.

None of the non-union culture samples showed evidence of microorganism growth. Bacterial isolates were found by conventional culturing methods in only 1 case of an open fracture from the control group (Figure 2). The isolated pathogens were *Streptococcus suis* and *Enterococcus* species. Tissue from the fracture gap in this case after initial débridement yielded negative results by PCR.

16 S rRNA was detected in tissue specimens from 2 cases (8.7%) of non-union. Further analysis of these variable species-specific sequences enabled the identification of specific microorganisms; in one case *Methylobacterium* species and in the other sample *Staphylococcus* species were identified. The PCR-positive patients were both culture-negative.

## Discussion

To the best of our knowledge, this is the first study on conventional and molecular bacteria detection methods in non-unions following tibia shaft fractures to validate the hypothesis that non-culturable microorganisms are a potential source of non-union.

Diaphyseal tibia fractures are the most common lower limb fractures worldwide [1]. There is controversy among the literature regarding the best way to manage open tibial shaft fractures. Recently, a metaanalysis of randomized prospective studies was performed comparing external fixators and unreamed IM nails. There was no statistically significant difference between the two methods of stabilization with respect to union, delayed union, deep infection and chronic osteomyelitis [26]. Non-union continues to be one of the orthopedist’s greatest challenges, especially in its septic form.

As for many other infectious processes, early detection can often alter the natural course of the disease and ultimately improve long-term outcomes for patients [27]. There might be clinical signs highly suggestive of infection, but diagnosis can be a difficult task, particularly in the case of late and/or chronic infections [28].

Laboratory markers such as C-reactive protein, erythrocyte sedimentation rate, and white blood count are sensitive markers of inflammation and plausible infection, but they are unable to localize the exact site of infection and they are associated with low specificity [29]. In this series, no significant difference was obvious between the treatment and control group according to inflammatory laboratory markers, although the mean values in both groups diverged from the norm. Elevations in patients from the control group might have been due to trauma, which often leads to increased levels of laboratory markers in the absence of infection [30,31].

The standard analyses for detecting microorganisms – gram staining (for microscopic investigation) and culturing of tissue biopsy specimens obtained during surgical revision – are reported to have poor sensitivity [12]. The sensitivity seems to be related to the amount of biopsies taken [32]. Antibiotics administered for perioperative prophylaxis, as well as extended transportation time, inadequate quantities of vital bacteria and preservation of specimens before processing may all lead to negative culture results [33].

Although there is a large body of evidence on wound bacteriology after open fractures [25] with positive culture results in up to 83% [34], the literature regarding infection in closed fracture gaps that affect healing is lacking [35]. In our series, none of the closed fractures in the control group were culture-positive.

Many molecular tools for bacterial DNA detection from clinical samples have been developed. One of the most significant contributions thus far has been amplification-based techniques (PCR), since some studies have confirmed its excellent sensitivity and specificity [27]. Moreover, the PCR technique takes less than 5 hours to complete, which is significantly shorter than the couple of days required for routine cultures. In a prospective study comparing PCR and culture techniques in the diagnosis of prosthetic joint infection, Gallo et al. demonstrated a significantly higher sensitivity, accuracy and negative predictive value for PCR versus culture [13]. There was 83% concordance between the results of intraoperative culture and PCR detection of causative bacteria [13]. This is in accordance with Hoeffel et al., who reported a PCR sensitivity and specificity of 71% and 49%, respectively, and a positive predictive value of 22% and a negative predictive value of 7%, when compared with culture methods [36]. The authors concluded that the PCR methods should not serve as screening tests for musculoskeletal infections, but they could be useful to confirm infections, especially after initiating antibiotic treatment. Shortcomings of the PCR technique compared to routine cultures are higher costs, false-positive results and problems with interpretation [27].

In our series, 16 S rRNA was detected in tissue specimens from 2 cases (8.7%) of non-union. In contrast to our results, Szczèsny et al. report the presence of bacteria in the callus of closed fractured bones in up to 42% [37]. Both viable bacteria and their DNA were detected. Interestingly, the majority of isolates were not detected in the fracture gap tissue but in the subcutaneous tissue and muscles. In patients with nonalignment, *S. epidermidis* and *aureus* were detected in 4 out of 24 patients, whereas in the delayed healing group bacterial isolates were found in 15 out of 43 patients [37]. The reasons for the diversity between their results and ours remain unclear, although one can pinpoint the fact that in the present study, tissue from non-unions was investigated. In addition, the results of 16 S PCR are known to be very susceptible for contamination. Our tests were performed under sterile conditions in a laboratory room designated for RNA isolation and identification only. Sodium chloride solution used for tissue samples was checked for the presence of 16 S rRNA and was found to be negative for all samples. Further, most authors consider the positive cultures of deep tissue specimens to be contamination from external sources [38]. Contamination of specimens could be precluded from being the source of detected isolates.

There are two limitations that need to be acknowledged and addressed regarding the present study. The first limitation concerns the heterogeneous patient population, which reflects the situation of patients with tibial non-union. The second limitation involves the extent to which the findings can be generalized beyond the cases studied. The number of cases is too small for broad generalizations. However, these limitations can be seen as fruitful avenues for future research along the same lines.

This is the first study comparing routine cultures and molecular bacterial DNA detection in tibial non-union. In summary, nonculturable pathogens seem to play a causative role in tibial non-unions. The combination of microbiological culture and broad-range PCR seems to substantially add to the number of microbiological diagnoses obtained and may improve the clinican’s ability to tailor therapy to the individual patient’s needs.

## Competing interests

The authors declare that they have no competing interests. None of the authors received financial support.

## Authors’ contributions

1. Conception and design of the study: JG, UG 2, Analysis and interpretation of data: SW, AP, AS, JG 3, Collection and assembly of data: SW, AP, UG, AS, JG 4, Drafting of the article: JG, UG. All authors read and approved the final manuscript.
